# Supplementary material for: ELMO2 is an essential regulator of carotid artery development
Source: Nat Commun. 2025 Jun 2;16:5108. doi: 10.1038/s41467-025-60105-9 (PMC12130350; doi:10.1038/s41467-025-60105-9)
Supplement: Supplementary file 1 — Supplementary Information [file 41467_2025_60105_MOESM1_ESM.pdf]

# ELMO2 is an essential regulator of carotid artery development

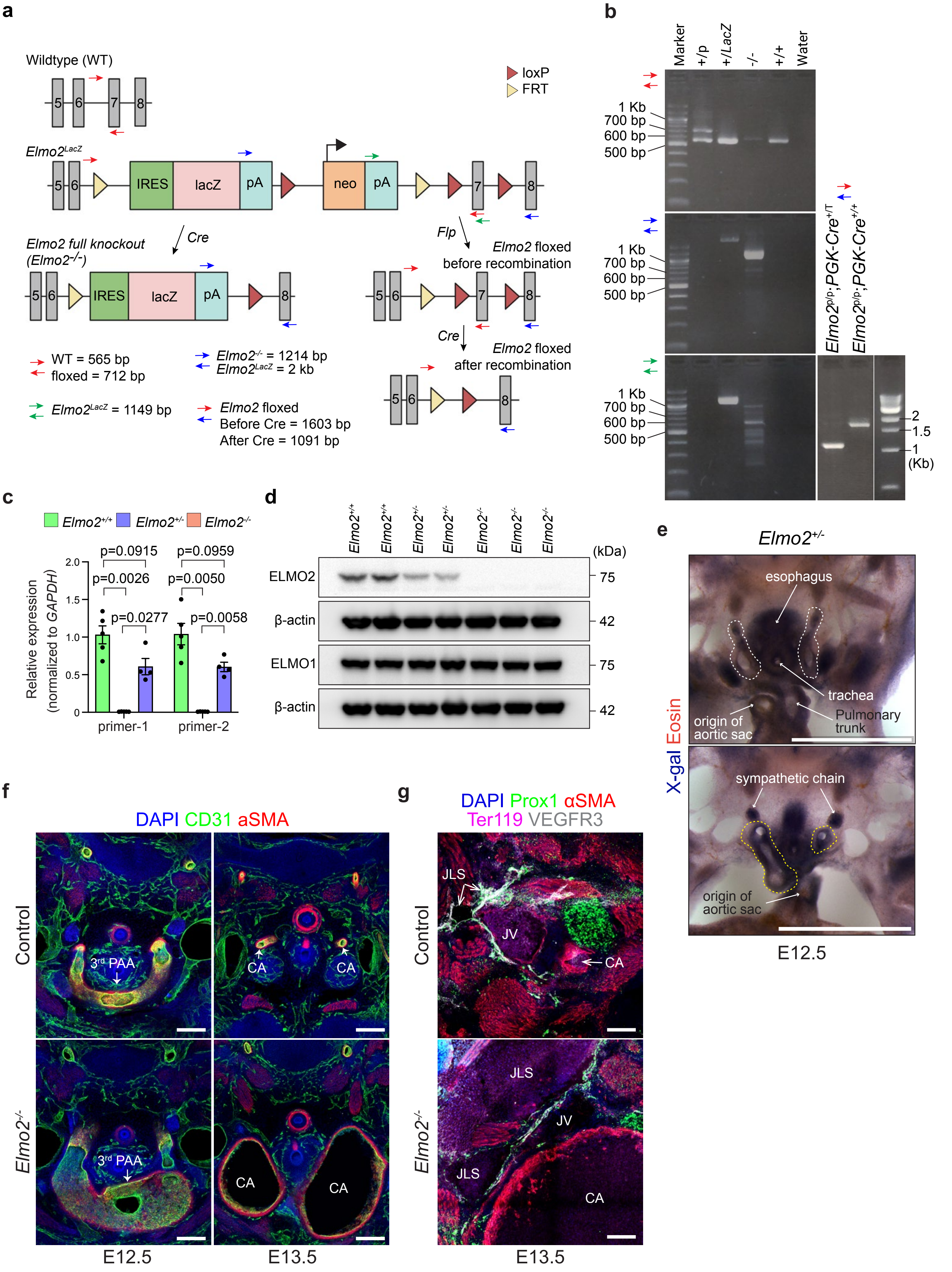


Suresh *et al.*, Supplementary Fig. 1

**Supplementary Figure 1. *Elmo2* knockout construct generation and validation**

**a)** *Elmo2* wildtype and targeted allele before and after Cre- or Flp-mediated recombination. Genotyping primers (colored arrows) and resulting amplicons are indicated. The *Elmo2* knockout first allele ($Elmo2^{LacZ}$) has an internal ribosome entry site (IRES) followed by *LacZ* and a polyadenylation signal (pA) upstream of exon 7. This allele yields a truncated, non-functional form of ELMO2 together with β-galactosidase. Global knockout mice ($Elmo2^{-/-}$) are obtained by breeding to homozygosity the offspring of $Elmo2^{LacZ}$ mice mated with *PGK-Cre* animals to ubiquitously delete exon 7 and the neomycin resistance gene (neo), which are flanked by loxP-sites (red triangles). The conditional knockout allele (*Elmo2* floxed) is obtained after Flp-mediated excision of sequences flanked by FRT-sites (yellow triangles). Breeding of the *Elmo2* floxed mice with tissue-specific Cre-drivers allows cell type-specific inactivation.

**b)** Genotyping amplicons (in basepairs, bp; or kilobases, Kb) using the primers indicated (**a**).

**c)** *Elmo2* expression (RT-qPCR) in E12.5 whole-embryo lysates of the indicated genotypes using different primer pairs. Mean ± SEM, n=5 ($Elmo2^{+/+}$ and $Elmo2^{-/-}$), n=4 ($Elmo2^{+/-}$). Brown-Forsythe and Welch ANOVA test.

**d)** Immunoblot of E12.5 whole-embryo lysates from the indicated genotypes. Molecular weight marker (kDa) is indicated.

**e)** Cross-section of E12.5 $Elmo2^{+/-}$ embryo stained with X-gal (blue) and Eosin (red) showing expression of *Elmo2* in cervical structures. Yellow and white dashed lines outline the 4$^{th}$ and 6$^{th}$ PAAs, respectively. Scale bars, 2mm.

**f)** Control and $Elmo2^{-/-}$ E12.5 and E13.5 cross-sections stained for nuclei (DAPI, blue), ECs (CD31, green) and VSMCs (αSMA, red) showing vascular dilation (3$^{rd}$ PAA) and carotid artery (CA) aneurysm in mutant embryos. Scale bars, 200µm.

**g)** Control and $Elmo2^{-/-}$ E13.5 cross-sections stained for nuclei (DAPI, blue), lymphatic ECs (Prox1, green; VEGFR3, grey), VSMCs (αSMA, red) and erythrocytes (Ter119, magenta). Note blood content in jugular lymph sac (JLS) and compressed jugular vein (JV) in addition to CA aneurysm in mutant embryos. Scale bars, 100µm.

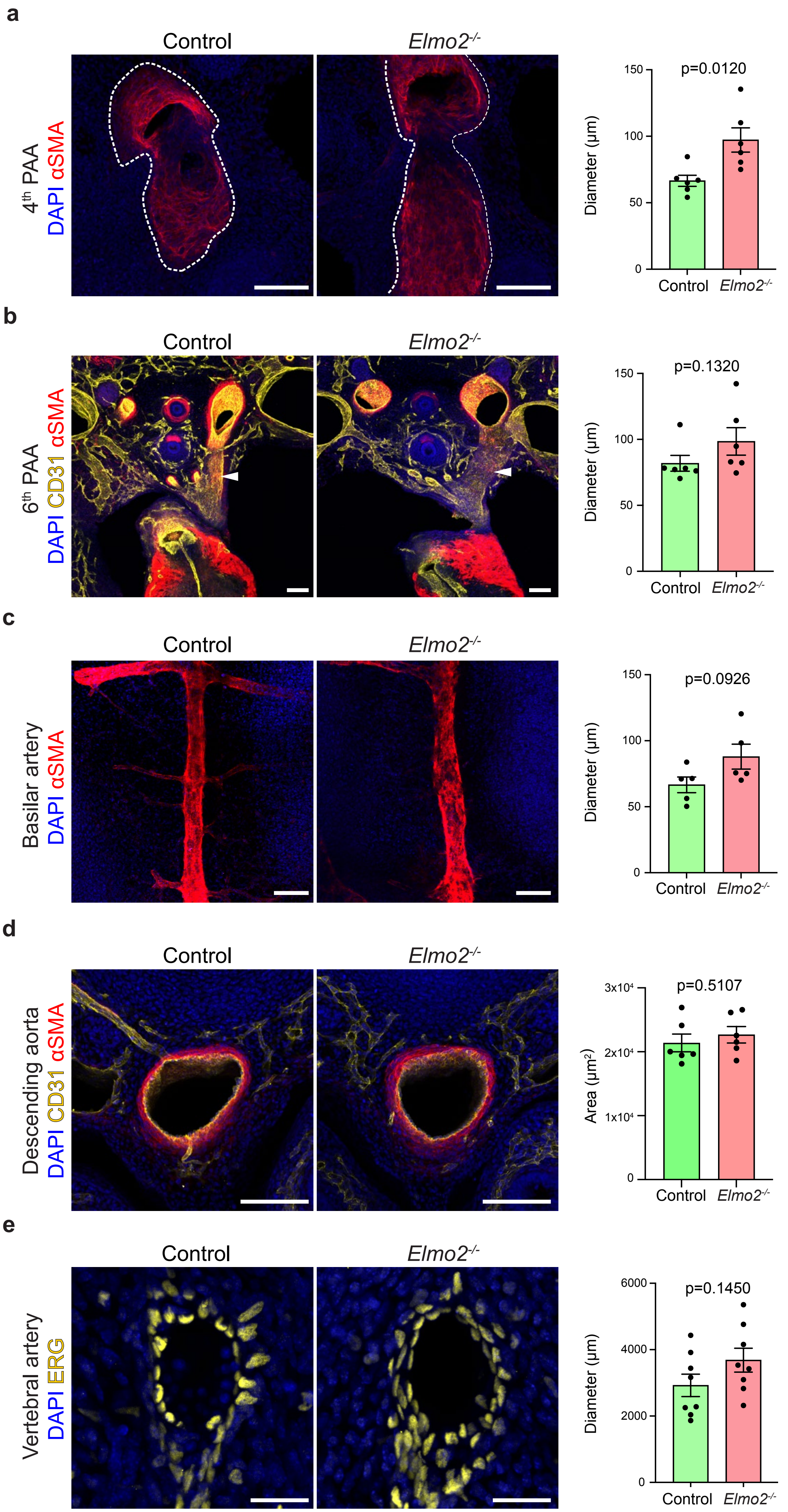


Suresh *et al.*, Supplementary Fig. 2

**Supplementary Figure 2. Vascular changes upon *Elmo2* inactivation**

**a)** Representative confocal image and diameter quantitation of the $4^{th}$ PAA (white dashed line) from control and *Elmo2*$^{-/-}$ E12.5 mouse embryos. Cross-sections stained for nuclei (DAPI, blue) and VSMCs (αSMA, red). Scale bars, 100µm. Bar plots: Mean ± SEM, n=6. Unpaired t-test.

**b)** Representative confocal image and diameter quantitation of the $6^{th}$ PAA (white arrowhead) from control and *Elmo2*$^{-/-}$ E12.5 mouse embryos. Cross-sections stained for nuclei (DAPI, blue), ECs (CD31, yellow) and VSMCs (αSMA, red). Scale bars, 100µm. Bar plots: Mean ± SEM, n=6. Mann-Whitney test.

**c)** Representative confocal image and diameter quantitation of the basilar artery from control and *Elmo2*$^{-/-}$ E12.5 mouse embryos. Cross-sections stained for nuclei (DAPI, blue) and VSMCs (αSMA, red). Scale bars, 100µm. Bar plots: Mean ± SEM, n=5. Unpaired t-test.

**d)** Representative confocal image and area quantitation of the descending aorta from control and *Elmo2*$^{-/-}$ E12.5 mouse embryos. Cross-sections stained for nuclei (DAPI, blue), ECs (CD31, yellow) and VSMCs (αSMA, red). Scale bars, 100µm. Bar plots: Mean ± SEM, n=6. Unpaired t-test.

**e)** Representative confocal image and diameter quantitation of the vertebral artery from control and *Elmo2*$^{-/-}$ E12.5 mouse embryos. Cross-sections stained for nuclei (DAPI, blue) and EC nuclei (ERG, yellow). Scale bars, 25µm. Bar plots: Mean ± SEM, n=8. Unpaired t-test.

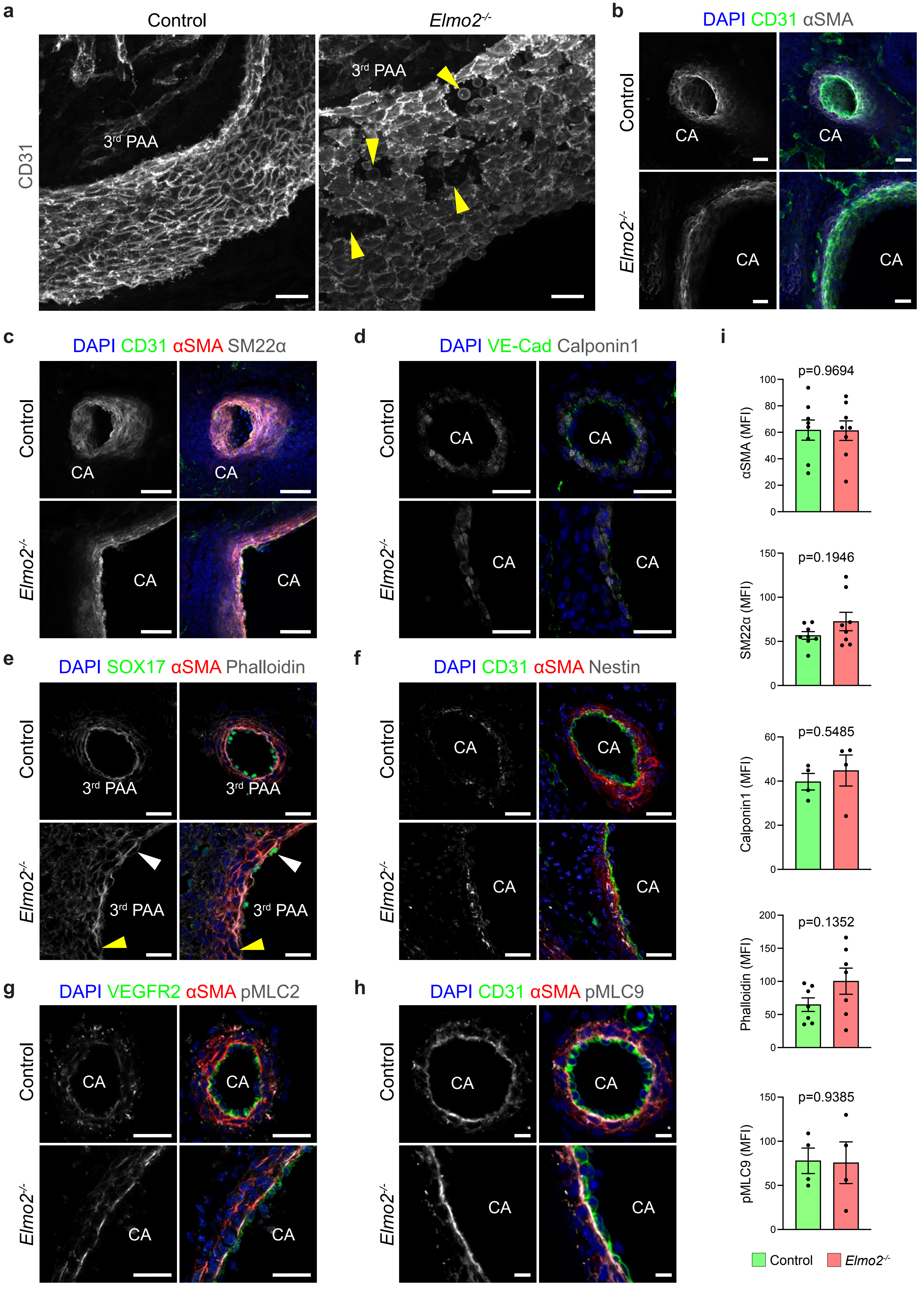


Suresh *et al.*, Supplementary Fig. 3

**Supplementary Fig. 3: Defects in endothelial and vascular smooth muscle cells of the third pharyngeal arch artery (3$^{rd}$ PAA) and carotid artery (CA) upon *Elmo2* global inactivation**

**a)** E12.5 control and *Elmo2*$^{-/-}$ 3$^{rd}$ PAA stained for ECs (CD31, grey). Yellow arrowheads point to abnormal discontinuities in the endothelial lining. Scale bars, 25µm.

**b)** E13.5 control and *Elmo2*$^{-/-}$ carotid arteries (CA) stained for nuclei (DAPI, blue), ECs (CD31, green) and VSMCs (αSMA, grey). Scale bars, 25µm.

**c)** E13.5 control and *Elmo2*$^{-/-}$ carotid arteries (CA) stained for nuclei (DAPI, blue), ECs (CD31, green) and VSMCs (αSMA, red; SM22α, grey). Scale bars, 25µm.

**d)** E13.5 control and *Elmo2*$^{-/-}$ carotid arteries (CA) stained for nuclei (DAPI, blue), EC junctions (VE-cadherin, green) and VSMCs (Calponin1, grey). Scale bars, 25µm.

**e)** E12.5 control and *Elmo2*$^{-/-}$ 3$^{rd}$ PAA stained for nuclei (DAPI, blue), arterial ECs (SOX17, green), VSMCs (αSMA, red) and actin (Phalloidin, grey). ECs with ectopic αSMA expression and discontinuous actin-ring in *Elmo2*$^{-/-}$ embryos are indicated by white and yellow arrowheads, respectively. Scale bars, 25µm.

**f)** E13.5 control and *Elmo2*$^{-/-}$ carotid arteries (CA) stained for nuclei (DAPI, blue), ECs (CD31, green) and VSMCs (αSMA, red; Nestin, grey). Scale bars, 25µm.

**g)** E13.5 control and *Elmo2*$^{-/-}$ carotid arteries (CA) stained for nuclei (DAPI, blue), ECs (VEGFR2, green), VSMCs (αSMA, red) and phosphorylated myosin light chain 2 (pMLC2, grey). Scale bars, 25µm.

**h)** E13.5 control and *Elmo2*$^{-/-}$ carotid arteries (CA) stained for nuclei (DAPI, blue), ECs (CD31, green), VSMCs (αSMA, red) and phosphorylated myosin light chain 9 (pMLC9, grey). Scale bars, 25µm.

**i)** Mean fluorescence intensity (MFI) for VSMC markers in immunostained cross-sections from E12.5 and E13.5 control and *Elmo2*$^{-/-}$ embryos. Mean ± SEM, n=8 (αSMA and SM22α), n=4 (Calponin1 and pMLC9), n=7 (Phalloidin). Welch's t-test (SM22α) and unpaired t-test (all other markers).

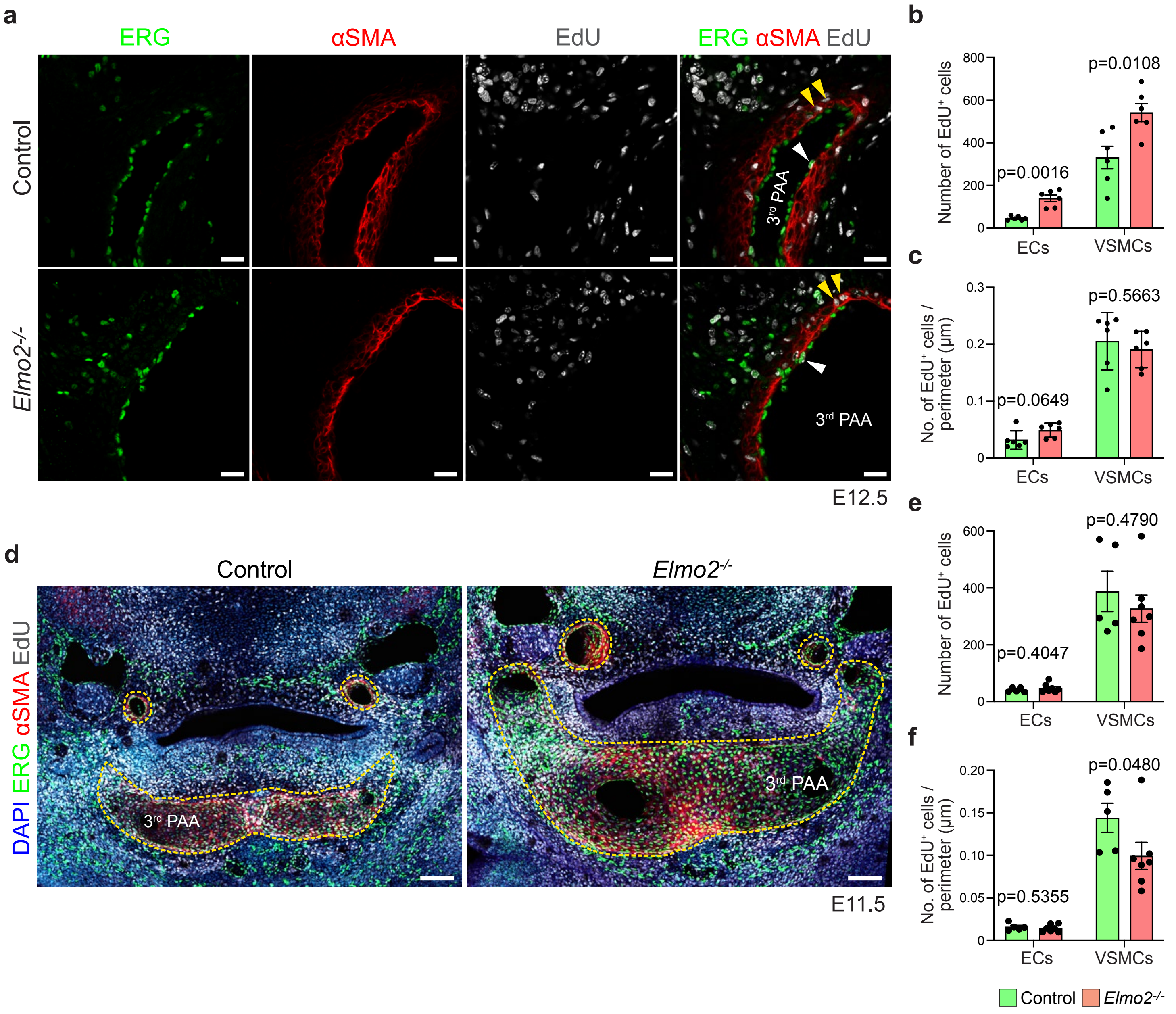


Suresh *et al.*, Supplementary Fig. 4

**Supplementary Figure 4. Analysis of embryonic cell proliferation**

**a)** High magnification confocal images of the third pharyngeal arch artery (3rd PAA) from control and *Elmo2*$^{-/-}$ E12.5 embryos stained for EC nuclei (ERG, green), VSMCs (αSMA, red) and proliferating cells (EdU, grey). White and yellow arrowheads point to dividing ECs and VSMCs, respectively. Scale bars, 25µm.

**b)** Total number of proliferating ECs and VSMCs in the 3rd PAA of control and *Elmo2*$^{-/-}$ E12.5 embryos. Mean ± SEM, n=8. Welch's t-test (ECs) and unpaired t-test (VSMCs).

**c)** Relative number of proliferating ECs and VSMCs normalized to vessel perimeter in the 3rd PAA of E12.5 control and *Elmo2*$^{-/-}$ embryos. Mean ± SEM, n=8. Mann-Whitney test (ECs) and unpaired t-test (VSMCs).

**d)** Overview confocal image of the 3rd PAA (yellow dashed line) from E11.5 control and *Elmo2*$^{-/-}$ embryos stained for nuclei (DAPI, blue), EC nuclei (ERG, green), VSMCs (αSMA, red) and proliferating cells (EdU, grey). Scale bars, 100µm.

**e)** Total number of proliferating ECs and VSMCs in the 3rd PAA of E11.5 control and *Elmo2*$^{-/-}$ embryos. Mean ± SEM, control (n=5), *Elmo2*$^{-/-}$ (n=7). Unpaired t-test.

**f)** Relative number of proliferating ECs and VSMCs normalized to vessel perimeter in the 3rd PAA of E11.5 control and *Elmo2*$^{-/-}$ embryos. Mean ± SEM, control (n=5), *Elmo2*$^{-/-}$ (n=7). Unpaired t-test (ECs) and Mann-Whitney test (VSMCs).

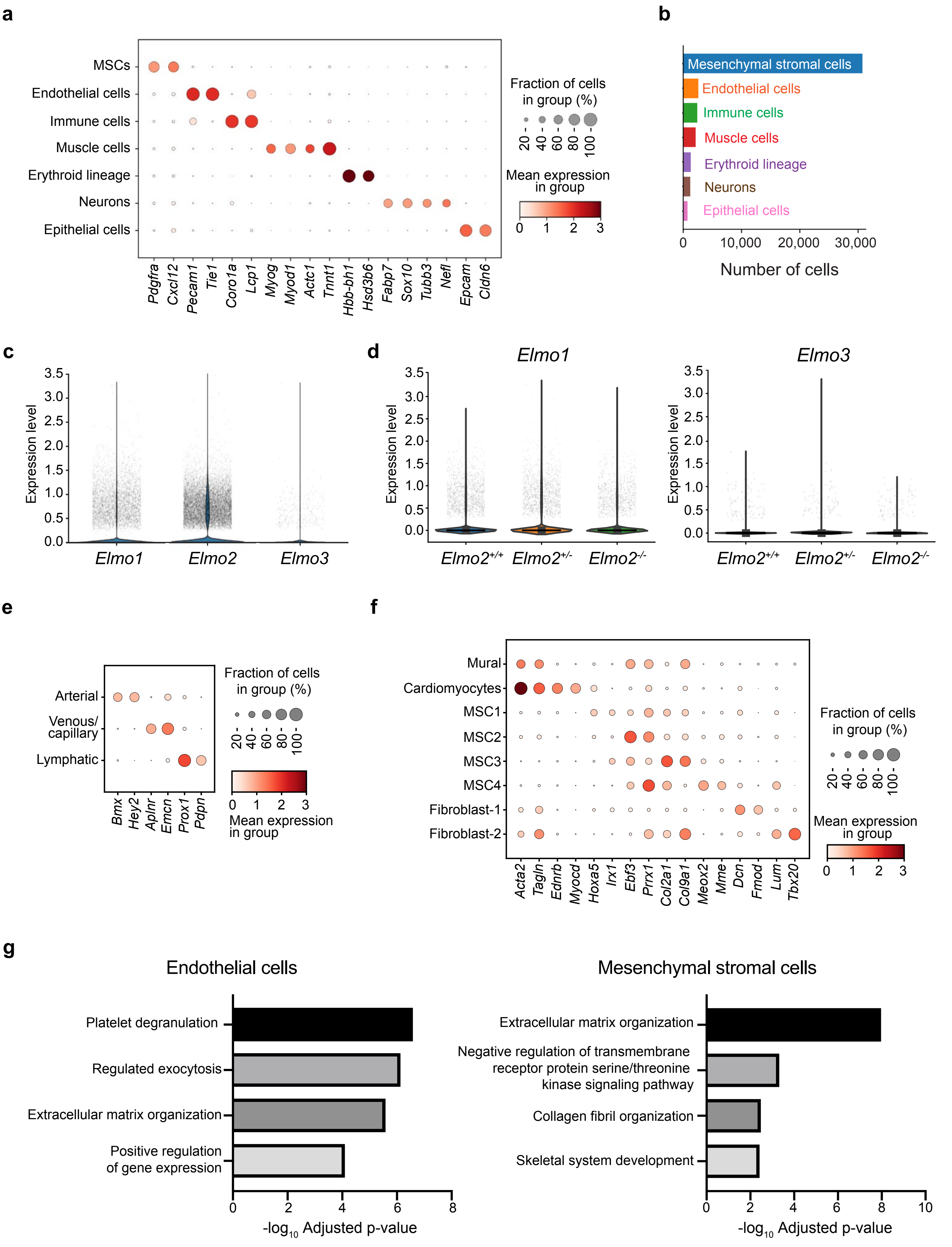


Suresh *et al*., Supplementary Fig. 5

**Supplementary Figure 5. Single-cell RNA sequencing data analysis**

**a)** Dot plot depicting the expression of cell type-specific genes within the different clusters identified in Fig. 3a. The fraction of cells expressing the gene with respect to the total population within the cluster is represented by the dot size and the mean expression within the group is color-coded.

**b)** Bar plot representation of the total number of cells in each cluster. Colors and cell identity correspond to those shown in Fig. 3a.

**c)** Violin plot showing the expression of *Elmo1*, *Elmo2* and *Elmo3* in the integrated data representing all cells analyzed.

**d)** Violin plot showing the expression of *Elmo1* and *Elmo3* within the different genotypes analyzed.

**e)** Dot plot showing the top cell type-specific genes within the endothelial cells subclusters as identified in Fig. 3d. The fraction of cells expressing the gene with respect to the total population within the subcluster is represented by the dot size and the mean expression within the group is color-coded.

**f)** Dot plot showing the top cell type-specific genes within the mesenchymal stromal cells subclusters as identified in Fig. 3h. The fraction of cells expressing the gene with respect to the total population within the subcluster is represented by the dot size and the mean expression within the group is color-coded.

**g)** Top gene ontology (GO) biological process terms enriched in de-regulated genes from *Elmo2*$^{-/-}$ endothelial and mesenchymal stromal cells compared to control. Selection of upregulated genes based on p-adjusted <0.01 and $\log_2$ fold change >0.5 or <-0.5. Fisher's exact test with Benjamini-Hochberg multiple testing correction.

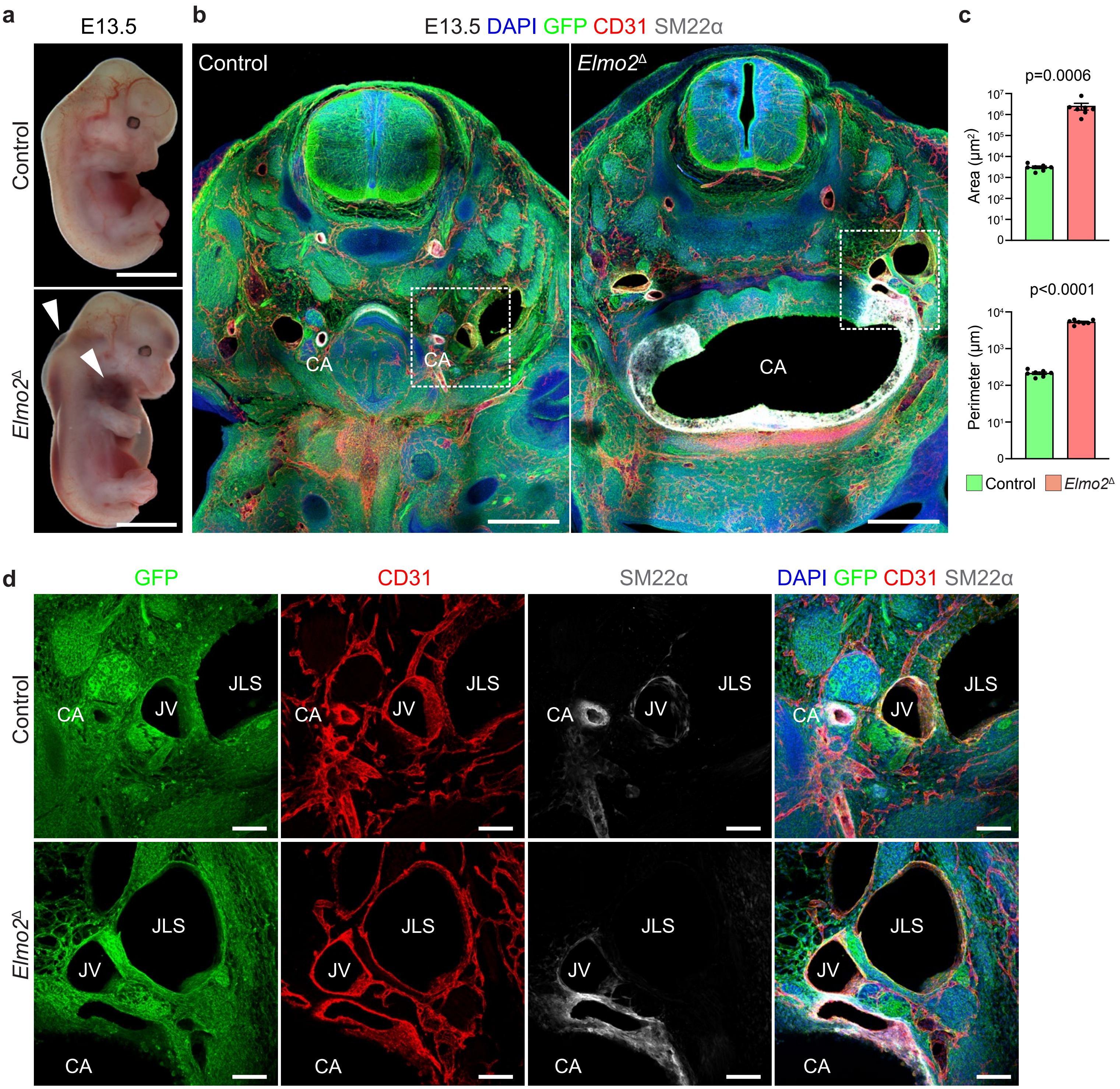


Suresh *et al.*, Supplementary. Fig. 6

**Supplementary Fig. 6: Functional validation of *Elmo2* floxed allele**

**a)** Representative images of control (*Elmo2*$^{+/p}$;*PGK-Cre*$^{+/T}$) and *Elmo2*$^{\Delta}$ (*Elmo2*$^{p/p}$;*PGK-Cre*$^{+/T}$) E13.5 embryos showing hemorrhages in the cervical region and subcutaneous edema (white arrowheads) in knockout mice. Scale bars, 2mm.

**b)** Confocal overview images of control and *Elmo2*$^{\Delta}$ E13.5 embryos stained for nuclei (DAPI, blue), recombined cells (GFP, green), ECs (CD31, red) and VSMCs (SM22α, grey) showing carotid artery (CA) aneurysm in knockout mice. Scale bars, 500μm.

**c)** Quantitation of carotid artery area and perimeter in E13.5 control and *Elmo2*$^{\Delta}$ embryos. Mean ± SEM, n=7. Mann-Whitney test (area) and Welch's t-test (perimeter).

**d)** Higher magnification images of dashed-line insets in (**b**) highlighting carotid arteries (CA) dilation and normal size of jugular vein (JV) and jugular lymph sac (JLS) in *Elmo2*$^{\Delta}$ embryos. Scale bars, 100μm.

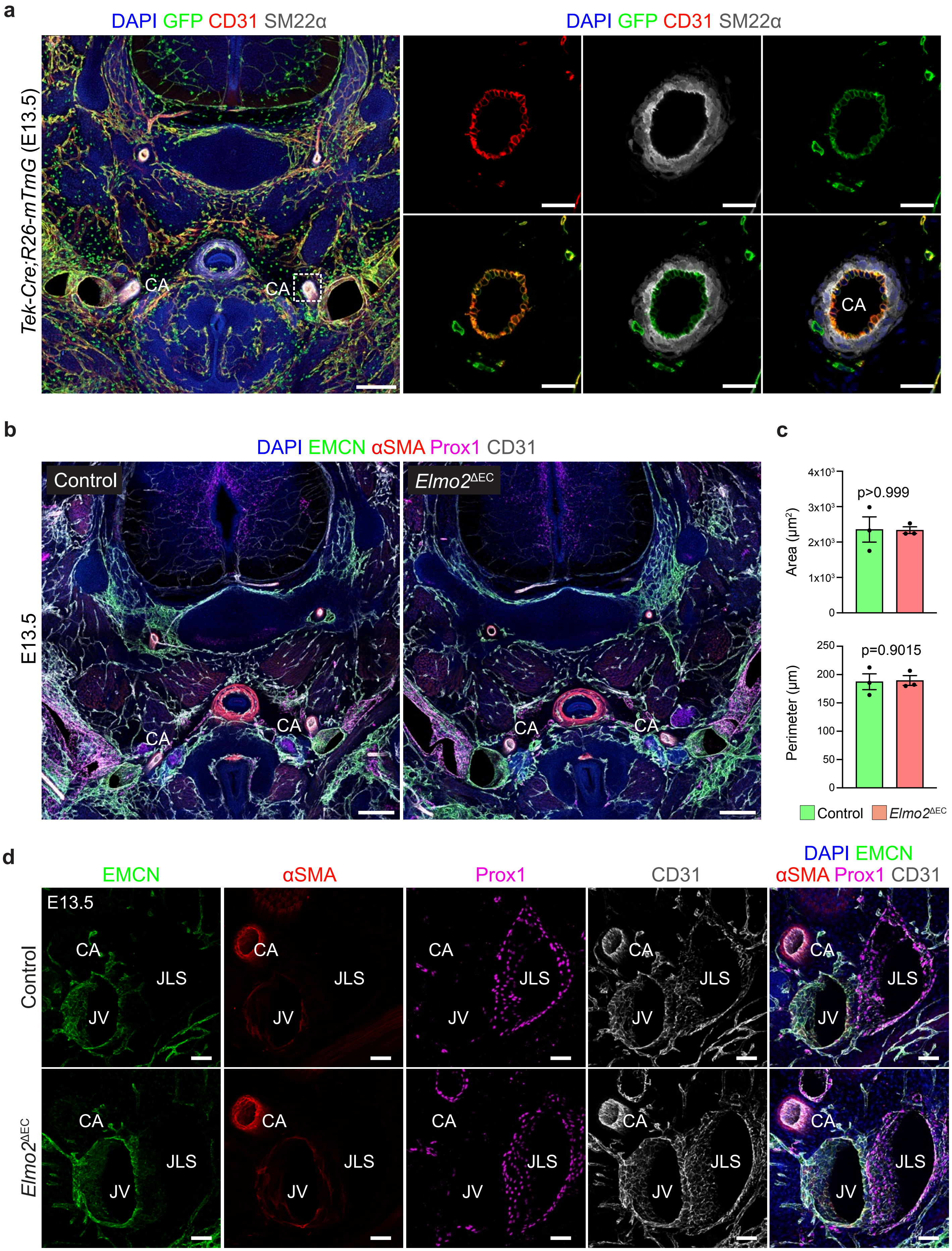


Suresh *et al.*, Supplementary Fig. 7

**Supplementary Figure 7. Endothelial cell-specific inactivation of *Elmo2***

**a)** Recombination analysis of *Tek-Cre;R26-mTmG* E13.5 embryos. Representative confocal images of the carotid artery (CA, dashed-line inset in overview image) stained for nuclei (DAPI, blue), recombined cells (GFP, green), ECs (CD31, red) and VSMCs (SM22α, grey). Higher magnification images on the right panel show GFP expression restricted to ECs. Scale bars, 200μm (overview) or 25μm (higher magnification).

**b)** Confocal overview images of cross-sections from the cervical region of E13.5 control (*Elmo2*$^{p/p}$;*Tek-Cre*$^{+/+}$) and *Elmo2*$^{\Delta EC}$ (*Elmo2*$^{p/p}$;*Tek-Cre*$^{+/T}$) embryos stained for nuclei (DAPI, blue), veins/capillaries (EMCN, green), VSMCs (αSMA, red), lymphatic ECs (Prox1, magenta) and ECs (CD31, grey). Note comparable diameter and morphology of the carotid artery (CA) in both samples. Scale bars, 200μm.

**c)** Quantitation of carotid artery area and perimeter in E13.5 control and *Elmo2*$^{\Delta EC}$ embryos. Mean ± SEM, n=3. Mann-Whitney test (area) or unpaired t-test (perimeter).

**d)** High magnification confocal images showing no overt changes in relevant vascular structures (CA, carotid artery; JV, jugular vein; JLS, jugular lymph sac) of E13.5 control and *Elmo2*$^{\Delta EC}$ embryos. Cross-sections stained for nuclei (DAPI, blue), veins/capillaries (EMCN, green), VSMCs (αSMA, red), lymphatic ECs (Prox1, magenta) and ECs (CD31, grey). Scale bars, 50μm.

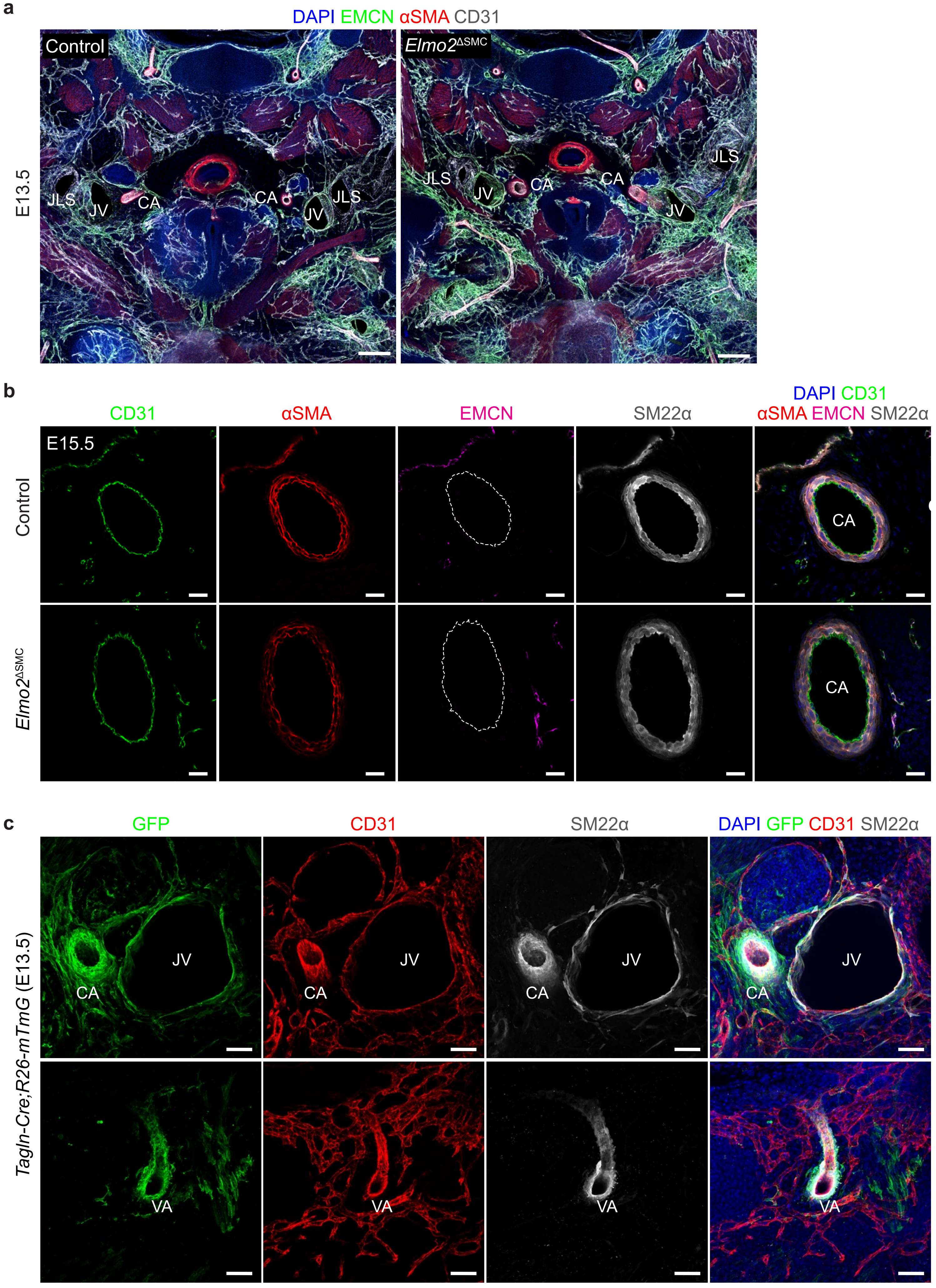


Suresh *et al.*, Supplementary Fig. 8

**Supplementary Figure 8. Smooth muscle cell-specific inactivation of *Elmo2***

**a)** Confocal overview images of smooth muscle cell-specific inactivation model of *Elmo2*. The cervical region from E13.5 control (*Elmo2*$^{p/p}$;*Tagln-Cre*$^{+/+}$) and *Elmo2*$^{\Delta SMC}$ (*Elmo2*$^{p/p}$;*Tagln-Cre*$^{+/T}$) embryos stained for nuclei (DAPI, blue), veins/capillaries (EMCN, green), VSMCs (αSMA, red) and ECs (CD31, grey). Note discrete dilation of the carotid arteries (CA) in *Elmo2*$^{\Delta SMC}$ embryos without overt changes in jugular vein (JV) or jugular lymph sac (JLS). Scale bars, 200μm.

**b)** High magnification confocal images of the carotid arteries (CA) from E15.5 control and *Elmo2*$^{\Delta SMC}$ embryos stained for nuclei (DAPI, blue), ECs (CD31, green), veins/capillaries (EMCN, magenta) and VSMCs (αSMA, red; SM22α, grey). Scale bars, 25μm.

**c)** Recombination analysis of *Tagln-Cre;R26-mTmG* E13.5 embryos. Representative confocal images of cross-sections from E13.5 embryos stained for nuclei (DAPI, blue), recombined cells (GFP, green), ECs (CD31, red) and VSMCs (SM22α, grey). Note that *Tagln*-Cre efficiently targets VSMCs around the carotid artery (CA), jugular vein (JV) and vertebral artery (VA). Scale bars, 50μm.

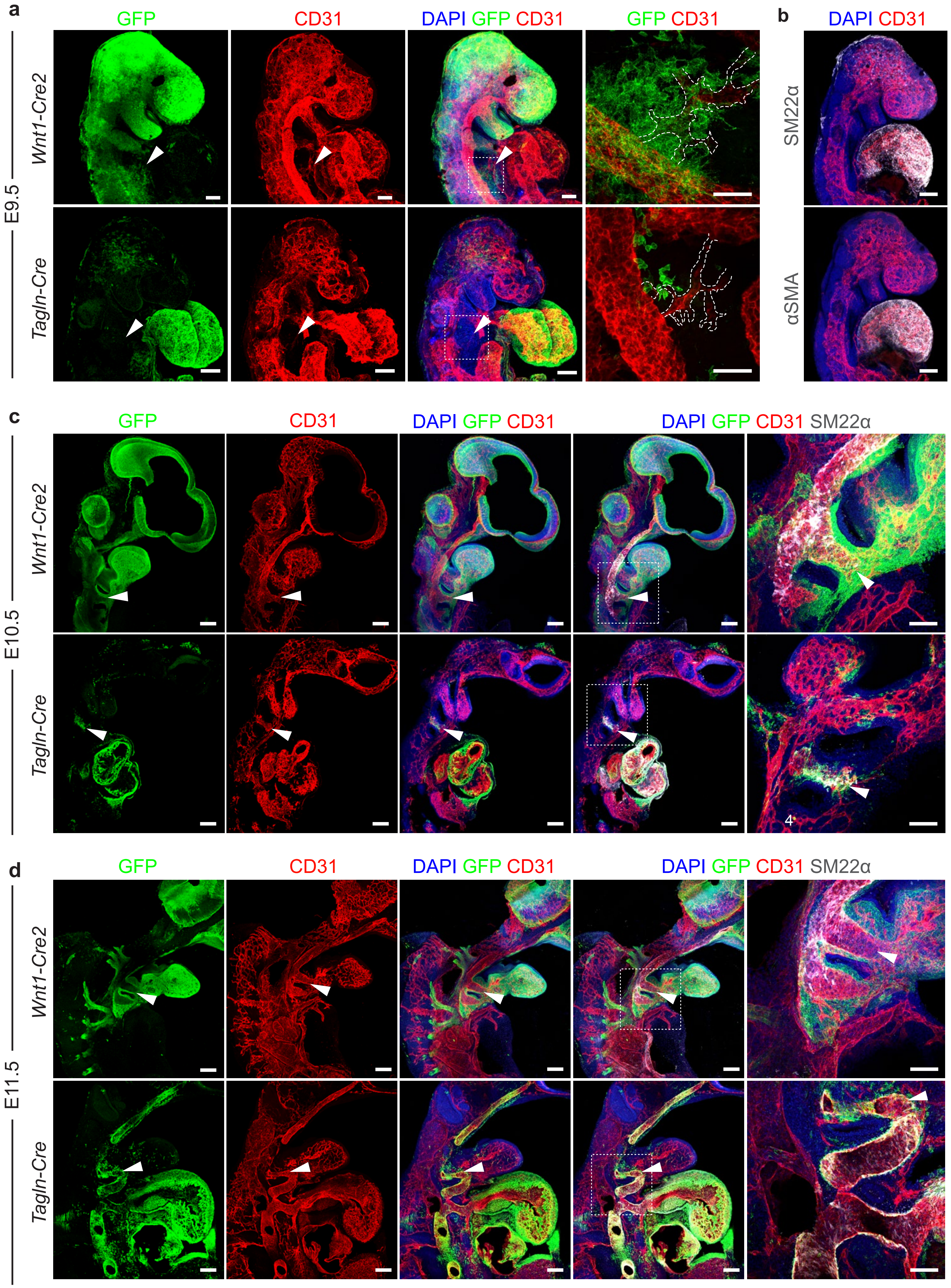


Suresh *et al.*, Supplementary Fig. 9

**Supplementary Figure 9. Comparative lineage tracing analysis for *Wnt1-Cre2* and *Tagln-Cre***

**a)** Confocal images of whole-mount E9.5 embryos stained for nuclei (DAPI, blue), recombined cells (GFP, green) and ECs (CD31, red). The vascular plexus giving rise to the 3$^{rd}$ PAA is indicated by a white arrowhead or outlined by a dashed line. High magnification views on the right correspond to the insets (white dash-line squares) shown in the preceding image and highlight the different targeting dynamics of perivascular mesenchymal cells with *Wnt1*- or *Tagln*-Cre in a *R26-mTmG*$^{+/p}$ background. Scale bars, 100µm (overview) and 50µm (higher magnification images).

**b)** Confocal images of whole-mount E9.5 embryos stained for nuclei (DAPI, blue) and VSMCs (SM22α and αSMA, grey). Scale bars, 100µm.

**c)** Confocal images of whole-mount E10.5 embryos stained for nuclei (DAPI, blue), recombined cells (GFP, green), ECs (CD31, red) and VSMCs (SM22α, grey). The 3$^{rd}$ PAA is indicated by white arrowheads. The high magnification views on the right correspond to the insets (white dash-line squares) shown in the preceding image and highlight differences in the abundance of recombined mesenchymal cells around the 3$^{rd}$ PAA. Scale bars, 100µm.

**d)** Confocal images of whole-mount E11.5 embryos stained for nuclei (DAPI, blue), recombined cells (GFP, green), ECs (CD31, red) and VSMCs (SM22α, grey). The 3$^{rd}$ PAA is indicated by a white arrowhead. The high magnification views on the right correspond to the insets (white dash-line squares) shown in the preceding image. Scale bars, 100µm.

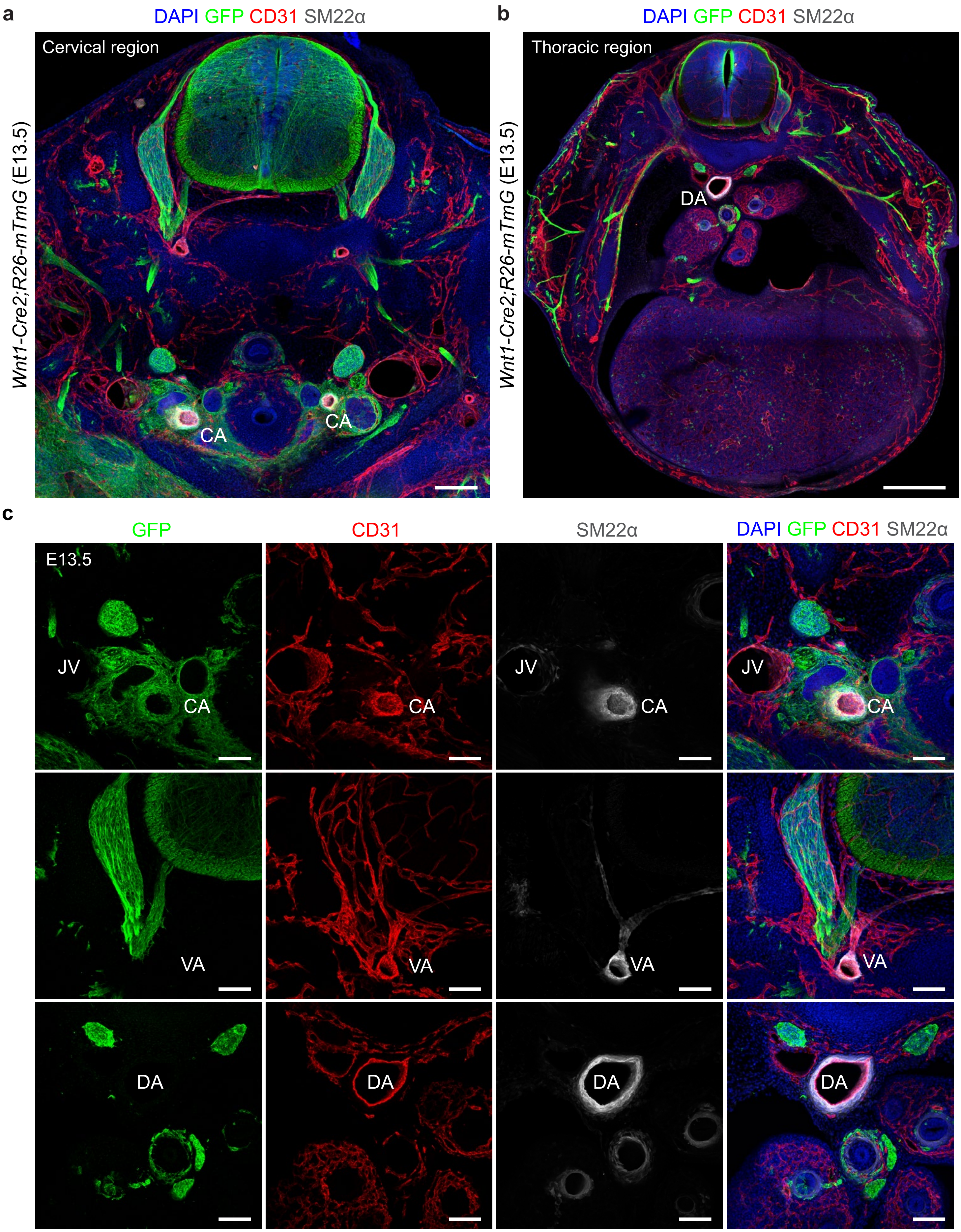


Suresh *et al.*, Supplementary Fig. 10

**Supplementary Figure 10**. **Lineage tracing analysis in *Wnt1-Cre2;R26-mTmG* embryos**

**a-b)** Representative overview confocal image from the cervical (**a**) or thoracic (**b**) region from E13.5 embryo cross-sections stained for nuclei (DAPI, blue), recombined cells (GFP, green), ECs (CD31, red) and VSMCs (SM22α, grey). Note high investment of $GFP^+$ cells around the carotid arteries (CA) but not around the descending aorta (DA). Scale bars, 200µm (**a**) and 500µm (**b**).

**c)** Higher magnification confocal images from E13.5 embryos stained for nuclei (DAPI, blue), recombined cells (GFP, green), ECs (CD31, red) and VSMCs (SM22α, grey) showing that GFP+ perivascular cells are only found around blood vessels with mural cells derived from neural crest origin, i.e. the carotid arteries (CA). Other major vessels, such as the jugular vein (JV), vertebral artery (VA) and descending aorta (DA) are covered by smooth muscle cells of a different ontogeny. Scale bars, 50µm.

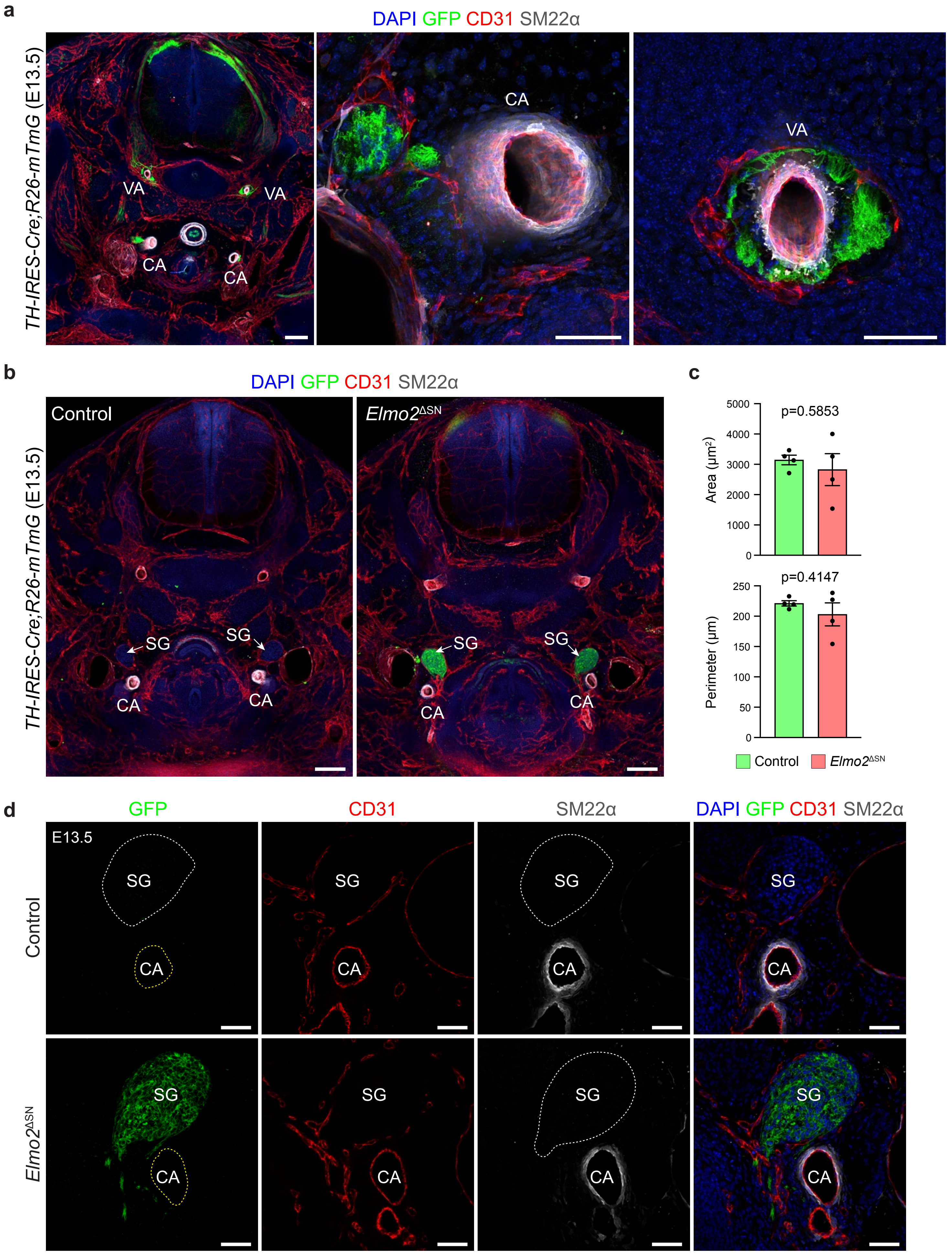


Suresh *et al.*, Supplementary Fig. 11

**Supplementary Figure 11. Inactivation of *Elmo2* in sympathetic neurons using *TH-IRES-Cre***

**a)** Recombination analysis in *TH-IRES-Cre;R26-mTmG* E13.5 embryos. Confocal images of the cervical region stained for nuclei (DAPI, blue), recombined cells (GFP, green), ECs (CD31, red) and VSMCs (SM22α, grey). Higher magnification images show expression of GFP in neural structures surrounding the carotid artery (CA) and vertebral artery (VA). Scale bars, 200μm (overview) and 50μm (higher magnification).

**b)** Confocal overview images of cross-sections from the cervical region of E13.5 control (*Elmo2*$^{p/p}$;*TH-IRES-Cre* $^{+/+}$) and *Elmo2*$^{\Delta SN}$ (*Elmo2*$^{p/p}$;*TH-IRES-Cre* $^{+/T}$) embryos stained for nuclei (DAPI, blue), recombined cells (GFP, green), ECs (CD31, red) and VSMCs (SM22α, grey). Note high degree of recombination in sympathetic ganglia (SG) without obvious changes in the diameter and morphology of the carotid artery (CA). Scale bars, 200μm.

**c)** Quantitation of area and perimeter of the carotid arteries from E13.5 control and *Elmo2*$^{\Delta SN}$ embryos. Mean ± SEM, n=4. Unpaired t-test (area) and Welch's t-test (perimeter).

**d)** High magnification confocal images showing no overt changes in carotid artery (CA, yellow dashed line) and the sympathetic ganglia (SG, white dashed line) of control and *Elmo2*$^{\Delta SN}$ E13.5 embryos. Cross sections stained for nuclei (DAPI, blue), recombined cells (GFP, green), ECs (CD31, red) and VSMCs (SM22α, grey). Scale bars, 50μm.

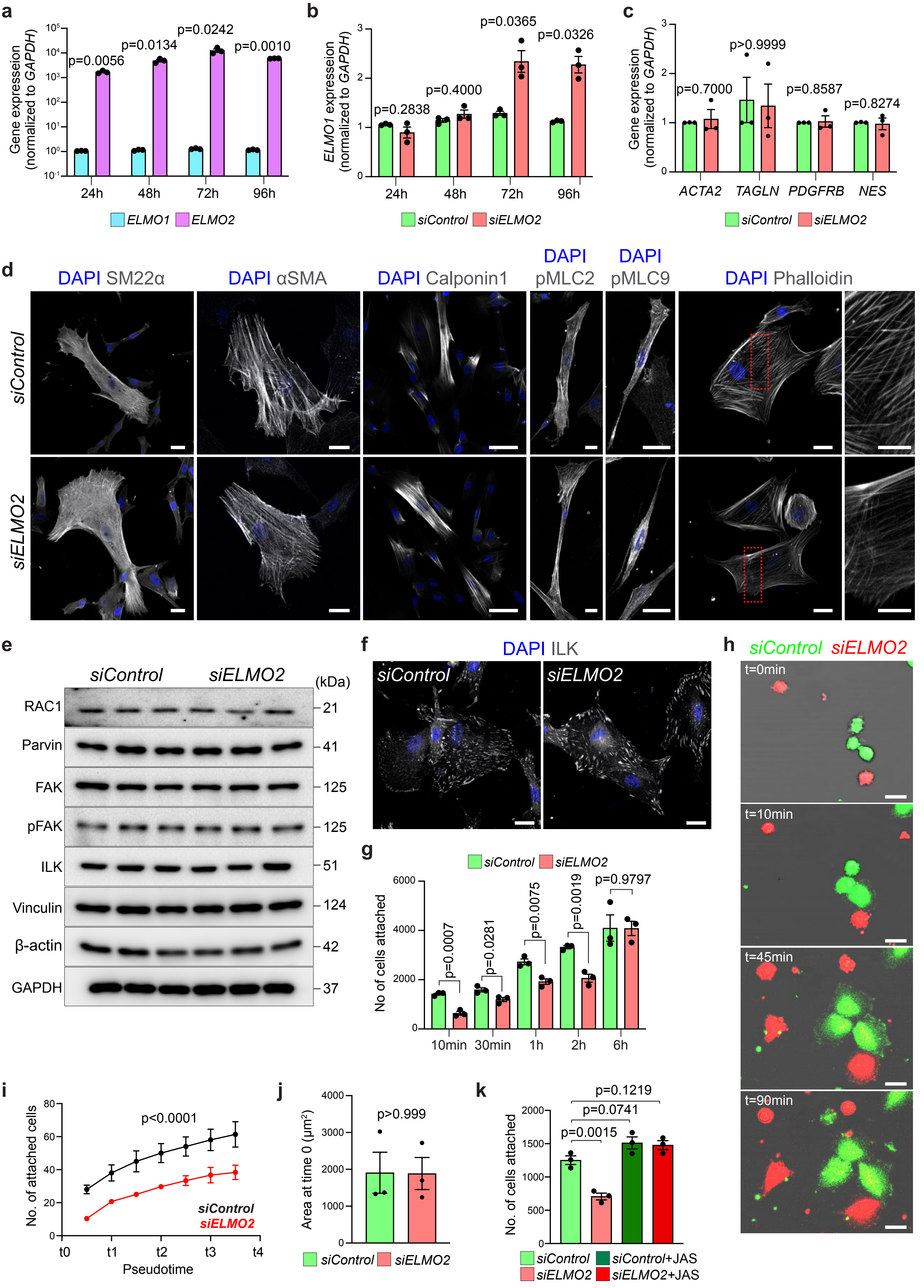


Suresh *et al.*, Supplementary Fig. 12

**Supplementary Figure 12. *In vitro* analysis of *ELMO2* knockdown in human brain vascular smooth muscle cells (HBVSMCs)**

**a)** *ELMO1* and *ELMO2* expression (RT-qPCR) in HBVSMCs. Mean ± SEM, n=3. Welch's t-test.

**b)** *ELMO1* expression (RT-qPCR) in *siControl* and *siELMO2* HBVSMCs at different timepoints (hours, h) after knockdown. Mean ± SEM, n=3. Welch's t-test (24 and 72h), Mann-Whitney (48h) and unpaired t-test (96h).

**c)** Expression (RT-qPCR) of VSMC markers 96h after *siControl* or *siELMO2* treatment. Mean ± SEM, n=3. Mann-Whitney test (*ACTA2* and *TAGLN*) and Welch's t-test (*PDGFRB* and *NES*).

**d)** Representative images of *siControl* and *siELMO2* HBVSMCs stained for nuclei (DAPI, blue) and VSMC or actin markers (SM22α, αSMA, Calponin1, phosphorylated myosin light chain 2 (pMLC2), phosphorylated myosin light chain 9 (pMLC9) and phalloidin, all in grey) 96h after knockdown. High magnification panels on the right, correspond to the insets (outlined by red dashed-line) on the Phalloidin staining and highlight the differences in stress fiber intensity between *siControl* and *siELMO2* cells. Scale bars, 25µm and 15µm (high magnification).

**e)** Immunoblot for ELMO2 molecular partners in lysates from *siControl* and *siELMO2* HBVSMCs 96h after knockdown. Molecular weight marker (kDa) is indicated.

**f)** Representative images of *siControl* and *siELMO2* HBVSMCs stained for nuclei (DAPI, blue), and integrin-linked kinase (ILK, grey) 96h after knockdown. Scale bars, 25µm.

**g)** Total number of *siControl* and *siELMO2* HBVSMCs attached at different timepoints along time-lapse cell spreading experiments. Mean ± SEM, n=3. Unpaired t-test.

**h)** Representative still frames from time-lapse cell spreading experiments. Cells are labeled with live-cell imaging reagents to differentiate between *siControl* (green) and *siELMO2* (red). Scale bars, 25µm.

**i)** Quantitative representation of adhesion dynamics of *siControl* and *siELMO2* HBVSMCs during time-lapse cell spreading experiments. Pseudotime calculated from seeding (t0). Mean ± SEM, n=3. Paired t-test.

**j)** Average cell area of *siControl* and *siELMO2* HBVSMCs before carbachol stimulation. Mean ± SEM, n=3. Mann-Whitney test.

**k)** Total number of cells attached after vehicle or jasplakinolide (JAS) treatment of *siControl* and *siELMO2* HBVSMCs. Mean ± SEM, n=3. Ordinary one-way ANOVA.

**Supplementary Table 1. Summary of *in vivo* phenotypes at E13.5 upon *Elmo2* global or tissue-specific deletion.**

| Mouse model | Target | TMX | CA dilation | CA aneurysm | EC defects | VSMC defects | Edema | Bleeding | Lethal |
|---|---|---|---|---|---|---|---|---|---|
| *Elmo2*$^{-/-}$ | Global | | X | X | X | X | X | X | X |
| *PGK-Cre*$^{+/T}$;*Elmo2*$^{p/p}$ | Global | | X | X | X | X | X | X | X |
| *R26-CreERT2* $^{+/T}$;*Elmo2*$^{p/p}$ | Global | E8.5-9.5 | X | X | X | X | X | X | X |
| *Wnt1-Cre2*$^{+/T}$;*Elmo2*$^{p/p}$ | NCCs | | X | X | X | X | X | X | X |
| *Tagln-Cre*$^{+/T}$;*Elmo2*$^{p/p}$ | VSMCs | | X | | | | | | |
| *R26-CreERT2* $^{+/T}$;*Elmo2*$^{p/p}$ | Global | E10.5-11.5 | X | | | | | | |
| *Tek-Cre* $^{+/T}$;*Elmo2*$^{p/p}$ | ECs | | | | | | | | |
| *TH-IRES-Cre*$^{+/T}$;*Elmo2*$^{p/p}$ | SNs | | | | | | | | |

Note: EC defects refer to abnormalities in polarity, monolayer continuity, retained Endomucin expression or ectopic expression of mesenchymal markers. VSMC defects refer to abnormal alignment of actin bundles and mislocalization of $\alpha$SMA and SM22$\alpha$ immunosignals. NCCs: Neural crest cells; VSMCs: Vascular smooth muscle cells; ECs: Endothelial cells; SNs: Sympathetic neurons; CA: Carotid arteries.

**Supplementary Table 2. Primary and Secondary Antibodies used in this study**

| Antibodies | Source | Identifier | Dilution |
|---|---|---|---|
| Chicken polyclonal anti-GFP | 2BScientific Ltd | Cat# GFP-1010 RRID:AB_2307313 | 1:500 |
| Chicken polyclonal anti-GFP | Abcam | Cat# ab13970 RRID:AB_300798 | 1:500 |
| Goat polyclonal anti-CD31 | R&D Systems | Cat# AF3628 RRID:AB_2161028 | 1:200 |
| Goat polyclonal anti-Podocalyxin | R&D Systems | Cat# AF1556 RRID:AB_354858 | 1:200 |
| Goat polyclonal anti-Sox17 | R&D Systems | Cat# AF1924 RRID:AB_355060 | 1:100 |
| Mouse monoclonal anti-Tubulin | Sigma | Cat# T5168 RRID:AB_477579 | 1:1000 |
| Mouse monoclonal anti-Alpha smooth muscle actin | Sigma | Cat# A2547 RRID:AB_476701 | 1:400 |
| Mouse monoclonal anti-Alpha smooth muscle actin-Cy3 conjugated | Sigma | Cat# C6198 RRID:AB_476856 | 1:400 |
| Mouse monoclonal anti-Alpha smooth muscle actin-FITC conjugated | Sigma | Cat# F3777 RRID:AB_476977 | 1:400 |
| Mouse monoclonal anti-Beta actin | Invitrogen | Cat# AM4302 RRID:AB_2536382 | 1:1000 |
| Mouse monoclonal anti-Rac1 | Millipore | Cat# 05-389 RRID:AB_309712 | 1:100 |
| Rabbit monoclonal anti-Calponin1 | Cell Signaling Technology | Cat# 17819 RRID:AB_2798789 | 1:100 |
| Rabbit monoclonal anti-Elmo1 | Cell Signaling Technology | Cat# 14457 RRID:AB_2798484 | 1:1000 |
| Rabbit monoclonal anti-ERG | Abcam | Cat# ab110639 RRID:AB_10864794 | 1:200 |
| Rabbit monoclonal anti-ILK | Abcam | Cat# ab76468 RRID:AB_2126930 | 1:1000 - WB<br>1:100 - ICC |
| Rabbit polyclonal anti-FAK | Cell Signaling Technology | Cat# 3285 RRID:AB_2269034 | 1:1000 |
| Rabbit polyclonal anti-phospho-FAK | Cell Signaling Technology | Cat# 3283 RRID:AB_2173659 | 1:1000 |
| Rabbit polyclonal anti-Phospho-Myosin Light Chain 2 | Cell Signaling Technology | Cat# 3671 RRID:AB_330248 | 1:100 |
| Rabbit polyclonal anti-Phospho-Myosin Light Chain 9 | ThermoFisher | Cat# PA1-26470 RRID:AB_795761 | 1:100 |
| Rabbit polyclonal anti-Elmo2 | ThermoFisher | Cat# PA5-28725 RRID:AB_2546201 | 1:1000 |
| Rabbit polyclonal anti-GAPDH | Cell Signaling Technology | Cat# #2118 RRID:AB_561053 | 1:5000 |
| Rabbit polyclonal anti-Parvin | Cell Signaling Technology | Cat# ; #4026 RRID:AB_2158936 | 1:1000 |
| Rabbit polyclonal anti-Prox-1 | ReliaTech | Cat# 102-PA32AG RRID:AB_10013821 | 1:100 |

| | | | |
|---|---|---|---|
| Rabbit polyclonal anti-SM22a | Abcam | Cat# ab14106<br>RRID:AB_443021 | 1:100 |
| Rabbit polyclonal anti-Vinculin | Proteintech | Cat#  26520-1-AP<br>RRID:AB_2868558 | 1:1000 |
| Rat monoclonal anti-Endomucin | Santa Cruz | Cat# SC-65495<br>RRID:AB_2100037 | 1:100 |
| Rat monoclonal anti-Nestin | Santa Cruz | Cat# sc-101541<br>RRID:AB_1126570 | 1:100 |
| Rat monoclonal anti-VE Cadherin | BD Biosciences | Cat# 555289<br>RRID:AB_395707 | 1:100 |
| Donkey anti-chicken Alexa flour 488 | Jackson Laboratories | Cat# 703-545-155 | 1:400 |
| Donkey anti-goat-Alexa Fluor 488 | Invitrogen | Cat# A11055 | 1:400 |
| Donkey anti-goat-Alexa Fluor 546 | Invitrogen | Cat# A11056 | 1:400 |
| Donkey anti-goat-Alexa Fluor 647 | Invitrogen | Cat# A21447 | 1:400 |
| Donkey anti-rabbit-Alexa Fluor 488 | Invitrogen | Cat# A21206 | 1:400 |
| Donkey anti-rabbit-Alexa Fluor 647 | Invitrogen | Cat# A31573 | 1:400 |
| Donkey anti-rat-Alexa Fluor 488 | Invitrogen | Cat# A21208 | 1:400 |
| Donkey anti-rat-Alexa Fluor Cy3 | Jackson Immuno Research | Cat# 712-165-153 | 1:400 |
| Goat anti rabbit IgG- HRP | Cell Signaling Technology | Cat# 7074 | 1:5000 |
| Sheep anti mouse IgG- HRP | Amersham | Cat# NA931 | 1:5000 |
