# Supplementary material for: ELMO2 is an essential regulator of carotid artery development
Source: Nat Commun. 2025 Jun 2;16:5108. doi: 10.1038/s41467-025-60105-9 (PMC12130350; doi:10.1038/s41467-025-60105-9)
Supplement: Supplementary file 3 — Description of Additional Supplementary Files [file 41467_2025_60105_MOESM3_ESM.pdf]

Description of Additional Supplementary Files

File Name: Supplementary Movie 1
Description: **Defects in cell attachment and spreading upon ELMO2 silencing** Representative video of siControl and siELMO2 HBVSMCs (96h post knockdown) attaching and spreading on Collagen I-coated wells. Note membrane blebbing and delayed attachment in ELMO2-knockdown cells. Temporal resolution is one frame per minute.
