# Supplementary material for: ELMO2 is an essential regulator of carotid artery development
Source: Nat Commun. 2025 Jun 2;16:5108. doi: 10.1038/s41467-025-60105-9 (PMC12130350; doi:10.1038/s41467-025-60105-9)
Supplement: Supplementary file 5 — Reporting Summary [file 41467_2025_60105_MOESM5_ESM.pdf]

nature portfolio

Corresponding author(s): Ralf H. Adams (lead contact)
Rodrigo Diéguez-Hurtado

Last updated by author(s): Apr 28, 2025

Reporting Summary

Nature Portfolio wishes to improve the reproducibility of the work that we publish. This form provides structure for consistency and transparency in reporting. For further information on Nature Portfolio policies, see our Editorial Policies and the Editorial Policy Checklist.

Statistics

For all statistical analyses, confirm that the following items are present in the figure legend, table legend, main text, or Methods section.

n/a	Confirmed	
☐	☒	The exact sample size (*n*) for each experimental group/condition, given as a discrete number and unit of measurement
☐	☒	A statement on whether measurements were taken from distinct samples or whether the same sample was measured repeatedly
☐	☒	The statistical test(s) used AND whether they are one- or two-sided<br>*Only common tests should be described solely by name; describe more complex techniques in the Methods section.*
☒	☐	A description of all covariates tested
☐	☒	A description of any assumptions or corrections, such as tests of normality and adjustment for multiple comparisons
☐	☒	A full description of the statistical parameters including central tendency (e.g. means) or other basic estimates (e.g. regression coefficient) AND variation (e.g. standard deviation) or associated estimates of uncertainty (e.g. confidence intervals)
☐	☒	For null hypothesis testing, the test statistic (e.g. *F*, *t*, *r*) with confidence intervals, effect sizes, degrees of freedom and *P* value noted<br>*Give P values as exact values whenever suitable.*
☒	☐	For Bayesian analysis, information on the choice of priors and Markov chain Monte Carlo settings
☒	☐	For hierarchical and complex designs, identification of the appropriate level for tests and full reporting of outcomes
☐	☒	Estimates of effect sizes (e.g. Cohen's *d*, Pearson's *r*), indicating how they were calculated

Our web collection on statistics for biologists contains articles on many of the points above.

Software and code

Policy information about availability of computer code

Data collection	ZEN software was used for acquisition of confocal microscopy images (Black edition, Zeiss) and experiments with live cells (Blue edition v2.6, Zeiss). Light-sheet images were acquired and processed using the imaging and deconvolution software Aurora (v0.5.0, M Squared Life). Data collection of RT-qPCR experiments was done with Bio-Rad CFX manager (v3.1). Western blots were imaged using Image Lab Touch Software (Bio Rad, ChemiDoc Imaging system). Rac1-activation G-LISA assay data was acquired using Gen5 Microplate reader and Imager software (v3.08, BioTek Instruments).
Data analysis	Vision 4D / arivis (v4.2, Zeiss) was used for light-sheet microscopy data analysis.<br>ImageJ (Fiji) was used for image processing, analysis and quantification.<br>Statistical analyses were performed with Graphpad Prism10 (v.10.2.1, Perkin Elmer).<br>Analysis of scRNA-seq data was performed using Python (3.8.2) and R (4.1.2) using the following packages: anndata (v0.8.0), gseapy (v0.10.8), h5py (v3.7.0), harmonypy (v0.0.5), leidenalg (v0.8.10), matplotlib (v3.8.4), numpy (v1.22.4), pandas (v1.4.2), pydeseq2 (v0.4.9), rpy2 (v3.5.2), scanpy (v1.9.1), scipy (v1.13.1), scrublet (v0.2.3), seaborn (v0.11.2), textalloc (v1.0.5) and umap-learn (v0.5.3).<br>The custom code used for scRNA-seq analysis in this study is based on existing packages and own contributions. It is deposited in a publicly available database and can be accessed through the following link: https://keeper.mpdl.mpg.de/d/c6072badc24b42e5b138/. Dependencies "scrna-tools" and "anndataview" can be found at https://github.com/Bioinformatics-Service-MPI-Munster.

For manuscripts utilizing custom algorithms or software that are central to the research but not yet described in published literature, software must be made available to editors and reviewers. We strongly encourage code deposition in a community repository (e.g. GitHub). See the Nature Portfolio guidelines for submitting code & software for further information.

Data

Policy information about availability of data

All manuscripts must include a data availability statement. This statement should provide the following information, where applicable:

- Accession codes, unique identifiers, or web links for publicly available datasets
- A description of any restrictions on data availability
- For clinical datasets or third party data, please ensure that the statement adheres to our policy

The single-cell RNA-seq data generated in this study is deposited in the NCBI GEO repository under accession number GSE278960. Any additional information required to reanalyze the data reported in this paper is available from the lead contact upon request. A Source Data file is provided with this paper. All other data supporting the findings of this study are available from the corresponding authors or reasonable request.

Research involving human participants, their data, or biological material

Policy information about studies with human participants or human data. See also policy information about sex, gender (identity/presentation), and sexual orientation and race, ethnicity and racism.

Reporting on sex and gender	N/A
Reporting on race, ethnicity, or other socially relevant groupings	N/A
Population characteristics	N/A
Recruitment	N/A
Ethics oversight	N/A

Note that full information on the approval of the study protocol must also be provided in the manuscript.

Field-specific reporting

Please select the one below that is the best fit for your research. If you are not sure, read the appropriate sections before making your selection.

☒ Life sciences ☐ Behavioural & social sciences ☐ Ecological, evolutionary & environmental sciences

For a reference copy of the document with all sections, see nature.com/documents/nr-reporting-summary-flat.pdf

Life sciences study design

All studies must disclose on these points even when the disclosure is negative.

Sample size	For in vivo experiments 3-8 embryos per experiment were used to account for littermate-specific or batch-specific effects.<br>For in vitro experiments 3-4 biological replicates were used in which each biological replicate is an independent experiment with its own technical replicates.
Data exclusions	No data was excluded from this study
Replication	Experiments were replicated at least 3 times or as stated in figure legends. Embryos used for in vivo data were obtained from at least two different pregnancies.
Randomization	In vivo samples/embryos used in this study were considered randomized, as they were collected from different litters on different days.<br>Downstream experiments were conducted on different batches at various time points to minimize batch effects.<br>Biological replicates of in vitro experiments were randomized by performing them independently in different batches at distinct time points.
Blinding	Blinding was not possible in this study due to the obvious phenotype observed in the mutant embryos. In order to minimize bias, randomization, standardized scoring criteria and quantitative readouts were used.

Reporting for specific materials, systems and methods

We require information from authors about some types of materials, experimental systems and methods used in many studies. Here, indicate whether each material, system or method listed is relevant to your study. If you are not sure if a list item applies to your research, read the appropriate section before selecting a response.

Materials & experimental systems

n/a	Involved in the study
	☒ Antibodies
	☒ Eukaryotic cell lines
☒	Palaeontology and archaeology
	☒ Animals and other organisms
☒	Clinical data
☒	Dual use research of concern
☒	Plants

Methods

n/a	Involved in the study
☒	ChIP-seq
☒	Flow cytometry
☒	MRI-based neuroimaging

Antibodies

Antibodies used

Chicken polyclonal anti-GFP 2BScientific Ltd Cat# GFP-1010 RRID:AB_2307313
Chicken polyclonal anti-GFP Abcam Cat# ab13970 RRID:AB_300798
Goat polyclonal anti-CD31 R&D Systems Cat# AF3628 RRID:AB_2161028
Goat polyclonal anti-Podocalyxin R&D Systems Cat# AF1556 RRID:AB_354858
Goat polyclonal anti-Sox17 R&D Systems Cat# AF1924 RRID:AB_355060
Mouse monoclonal anti- Tubulin Sigma Cat# T5168 RRID:AB_477579
Mouse monoclonal anti-Alpha smooth muscle actin Sigma Cat# A2547 RRID:AB_476701
Mouse monoclonal anti-Alpha smooth muscle actin-Cy3 conjugated Sigma Cat# C6198 RRID:AB_476856
Mouse monoclonal anti-Alpha smooth muscle actin-FITC conjugated Sigma Cat# F3777
RRID:AB_476977
Mouse monoclonal anti-Beta actin Invitrogen Cat# AM4302 RRID:AB_2536382
Mouse monoclonal anti-Rac1 Millipore Cat# 05-389, RRID:AB_309712
Rabbit monoclonal anti-Calponin1 Cell Signaling Cat# 17819, RRID:AB_2798789
Rabbit monoclonal anti-Elmo1 Cell Signaling Cat# 14457 RRID:AB_2798484
Rabbit monoclonal anti-ERG Abcam Cat# ab110639 RRID:AB_10864794
Rabbit monoclonal anti-ILK Abcam Cat# ab76468, RRID:AB_2126930
Rabbit polyclonal anti-FAK Cell Signaling Cat# 3285, RRID:AB_2269034
Rabbit polyclonal anti- phospho-FAK Cell Signaling Cat# 3283, RRID:AB_2173659
Rabbit polyclonal anti- Phospho-Myosin Light Chain 2 Cell Signaling Cat# 3671, RRID:AB_330248
Rabbit polyclonal anti-Elmo2 ThermoFisher Cat# PA5-28725 RRID:AB_2546201
Rabbit polyclonal anti-GAPDH Cell Signaling Cat# #2118, RRID:AB_561053
Rabbit polyclonal anti-Parvin Cell Signaling Cat# ; #4026, RRID:AB_2158936
Rabbit polyclonal anti-Prox-1 ReliaTech Cat# 102-PA32AG RRID:AB_10013821
Rabbit polyclonal anti-Phospho-Myosin Light Chain 9 ThermoFisher Cat#Cat# PA1-26470 RRID:AB_795761
Rabbit polyclonal anti-SM22a Abcam Cat# ab14106 RRID:AB_443021
Rabbit polyclonal anti-Vinculin Proteintech Cat# 26520-1-AP RRID:AB_2868558
Rat monoclonal anti-Endomucin Santa Cruz Cat# SC-65495 RRID:AB_2100037
Rat monoclonal anti-Nestin Santa Cruz Cat# sc-101541, RRID:AB_1126570
Rat monoclonal anti-VE Cadherin BD Biosciences Cat# 555289 RRID:AB_395707
Donkey anti-chicken Alexa flour 488 Jackson Laboratories Cat# 703-545-155
Donkey anti-goat-Alexa Fluor 488 Invitrogen Cat# A11055
Donkey anti-goat-Alexa Fluor 546 Invitrogen Cat# A11056
Donkey anti-goat-Alexa Fluor 647 Invitrogen Cat# A21447
Donkey anti-rabbit-Alexa Fluor 488 Invitrogen Cat# A21206
Donkey anti-rabbit-Alexa Fluor 647 Invitrogen Cat# A31573
Donkey anti-rat-Alexa Fluor 488 Invitrogen Cat# A21208
Donkey anti-rat-Alexa Fluor Cy3 Jackson Immuno Research Cat# 712-165-153
Goat anti rabbit IgG- HRP Cell Signaling Cat# 7074
Sheep anti mouse IgG- HRP Amersham Cat# NA931

Validation

Chicken polyclonal anti-GFP (Abcam) Validated for the following by the supplier - Western blotting and immunofluorescence Reactivity- mouse Dilution 1:500 for IF vibratome and cryo sections
Chicken polyclonal anti-GFP Validated for the following by the supplier - ELISA, Immunocytochemistry, Immunohistochemistry, Western Blot Reactivity- mouse Dilution 1:500 for IF vibratome and cryo sections
Goat polyclonal anti-CD31 Validated for the following by the supplier - Western blotting, ELISA and immunofluorescence Reactivity- Human, mouse, rat Dilution 1:200 for IF vibratome and cryo sections
Goat polyclonal anti-Podocalyxin Validated for the following by the supplier - Western blotting, ELISA and immunofluorescence Reactivity- mouse Dilution 1:200 for IF vibratome and cryo sections
Goat polyclonal anti-Sox17 Validated for the following by the supplier - Western blotting, ELISA and immunofluorescence Reactivity- Human, mouse Dilution 1:100 for IF vibratome and cryo sections
Mouse monoclonal anti- Tubulin Validated for the following by the supplier - Western blotting, immunolabelling cells in electron microscopy and immunofluorescence Reactivity- human, mouse, rat Dilution 1:1000 for Western blotting

Mouse monoclonal anti-Alpha smooth muscle actin Validated for the following by the supplier - immunohistochemistry (formalin-fixed, paraffin-embedded sections): suitable using smooth muscl

Rabbit polyclonal anti-Phospho-Myosin Light Chain 9 (ThermoFisher) Validated for the following by the supplier - Western blotting (Dilution- 1:500-1:2,000), immunohistochemistry- (Dilution- 2-5ug/mL), ELISA (Dilution- 1:10,000-1:30,000) and immunoprecipitation (Dilution- 1:100). Validated in this study- immunohistochemistry in vibratome sections of mouse embryos and cultured cells of human origin with Dilution 1:100 immunohistochemistry (frozen sections): suitable using smooth muscle cells

indirect immunofluorescence: 1:400 using blood vessels in sections of human appendix

western blot: suitable using smooth muscle cells Reactivity- human, mouse Dilution 1:400 for Western blotting

Mouse monoclonal anti-Alpha smooth muscle actin-Cy3 conjugated Validated for the following by the supplier - immunofluorescence Reactivity- human, mouse Dilution 1:400 for IF vibratome & cryo sections, 1:100- immunocytochemistry

Mouse monoclonal anti-Alpha smooth muscle actin-FITC conjugated Validated for the following by the supplier - immunohistochemistry (formalin-fixed, paraffin-embedded sections Reactivity- human, mouse Dilution 1:400 for IF vibratome and cryo sections

Mouse monoclonal anti-Beta actin Validated for the following by the supplier - Western blot Reactivity- human, rabbit, mouse Dilution 1:1000 for Western blotting

Mouse monoclonal anti-Rac1 Validated for the following by the supplier – Immunohistochemistry, Immunoprecipitation, and Western Blotting Reactivity- human, mouse Dilution 1:100 for Western blotting

Rabbit monoclonal anti-Calponin1 Validated for the following by the supplier – Immunohistochemistry, immunofluorescence Immunoprecipitation, and Western Blotting Reactivity- human, mouse, rat Dilution 1:100 for IF vibratome sections, immunocytochemistry

Rabbit monoclonal anti-Elmo1 Validated for the following by the supplier – immunoprecipitation and Western Blotting Reactivity- human Dilution 1:1000 for Western blotting

Rabbit monoclonal anti-ERG Validated for the following by the supplier – Immunohistochemistry (P), immunofluorescence, immunocytochemistry Immunoprecipitation, Western Blotting and FACS Reactivity- human, mouse, Dilution 1:200 for IF vibratome and cryo sections

Rabbit monoclonal anti-ILK Validated for the following by the supplier – Immunohistochemistry (P), immunofluorescence, immunocytochemistry Immunoprecipitation, and Western Blotting, FACS Reactivity- human, mouse, rat Dilution 1:100 for immunocytochemistry, 1:1000 for Western blotting

Rabbit polyclonal anti-FAK Validated for the following by the supplier – Immunohistochemistry, Immunoprecipitation, and Western Blotting Reactivity- human, mouse, rat Dilution 1:1000 for Western blotting

Rabbit polyclonal anti- phospho-FAK Validated for the following by the supplier – Western Blotting Reactivity- human, mouse, rat Dilution 1:1000 for Western blotting

Rabbit polyclonal anti- Phospho-Myosin Light Chain 2 Validated for the following by the supplier – Western Blotting & immunofluorescence Reactivity- human, mouse, rat Dilution 1:100 vibratome sections, immunocytochemistry

Rabbit polyclonal anti-Elmo2 Validated for the following by the supplier – Western Blotting Reactivity- human, mouse, rat Dilution 1:1000 for Western blotting

Rabbit polyclonal anti-GAPDH Validated for the following by the supplier – Western Blotting, FACS, immunofluorescence, immunocytochemistry Reactivity- human, mouse, rat Dilution 1:5000 for Western blotting

Rabbit polyclonal anti-Parvin Validated for the following by the supplier – Western Blotting & immunofluorescence Reactivity- human, mouse, rat Dilution 1:1000 for Western blotting

Rabbit polyclonal anti-Prox-1 Validated for the following by the supplier – Western Blotting & immunofluorescence Reactivity- human, mouse Dilution 1:100 for IF vibratome & cryo sections

Rabbit polyclonal anti-SM22a Validated for the following by the supplier – Western Blotting & immunofluorescence Reactivity- human, mouse Dilution 1:100 for IF vibratome sections, immunocytochemistry

Rabbit polyclonal anti-Vinculin Validated for the following by the supplier – Western Blotting & immunofluorescence Reactivity- human, mouse, rat Dilution 1:1000 for Western blotting

Rat monoclonal anti-Endomucin Validated for the following by the supplier – Western Blotting & immunofluorescence Reactivity- mouse Dilution 1:100 for IF vibratome & cryo sections

Rat monoclonal anti-Nestin Validated for the following by the supplier – Western Blotting & immunofluorescence Reactivity- mouse Dilution 1:100 for IF vibratome sections

Rat monoclonal anti-VE Cadherin Validated for the following by the supplier – immunofluorescence, immunocytochemistry Reactivity- mouse Dilution 1:100 for IF vibratome & cryo sections

Donkey anti-chicken Alexa flour 488 Validated for the following by the supplier – immunofluorescence, immunocytochemistry Dilution 1:400 for IF vibratome and cryo sections

Donkey anti-goat-Alexa Fluor 488 Validated for the following by the supplier – immunofluorescence, immunocytochemistry, FACS Dilution 1:400 for IF vibratome and cryo sections

Donkey anti-goat-Alexa Fluor 546 Validated for the following by the supplier – immunofluorescence, immunocytochemistry Dilution 1:400 for IF vibratome and cryo sections

Donkey anti-goat-Alexa Fluor 647 Validated for the following by the supplier – immunofluorescence, immunocytochemistry Dilution 1:400 for IF vibratome and cryo sections

Donkey anti-rabbit-Alexa Fluor 488 Validated for the following by the supplier – immunofluorescence, immunocytochemistry Dilution 1:400 for IF vibratome and cryo sections

Donkey anti-rabbit-Alexa Fluor 647 Validated for the following by the supplier – immunofluorescence, immunocytochemistry, Immunoblot Dilution 1:400 for IF vibratome and cryo sections

Donkey anti-rat-Alexa Fluor 488 Validated for the following by the supplier – immunofluorescence, immunocytochemistry Dilution 1:400 for IF vibratome and cryo sections

Donkey anti-rat-Alexa Fluor Cy3 Validated for the following by the supplier – immunofluorescence, immunocytochemistry Dilution 1:400 for IF vibratome and cryo sections

Goat anti rabbit IgG- HRP Validated for the following by the supplier – Western Blotting Dilution 1:5000 for Western blotting

Sheep anti mouse IgG- HRP Validated for the following by the supplier – Western Blotting Dilution 1:5000 for Western blotting

Eukaryotic cell lines

Policy information about cell lines and Sex and Gender in Research

Cell line source(s)	Human brain vascular smooth muscle cells (HBVSMCs) were obtained as passage 0 (P0) from ScienCell, cat. #1100
Authentication	Cells were assessed for the expression of known identity markers and their contractile-differentiation capacity was confirmed by qPCR, Western blot and immunostaining.
Mycoplasma contamination	Cells routinely tested negative for mycoplasma contamination.
Commonly misidentified lines (See ICLAC register)	The cell line used in this study is not listed in the ICLAC database.

Animals and other research organisms

Policy information about studies involving animals; ARRIVE guidelines recommended for reporting animal research, and Sex and Gender in Research

Laboratory animals	Mice (Mus musculus). All genetically modified mouse lines are described in the Methods section of the study.
Wild animals	Study did not involve wild animals
Reporting on sex	Sex was not used as a variable in this study
Field-collected samples	N/A
Ethics oversight	Animals used in this study were housed in the animal house of the Max Planck Institute for Molecular Biomedicine, Münster. All animal procedures were conducted in accordance with the guidelines of the Max Planck Institute and approved by the the Landesamt für Natur, Umwelt und Verbraucherschutz Nordrhein-Westfalen (LANUV, Az. No. 81-02.04.2023.A383).

Note that full information on the approval of the study protocol must also be provided in the manuscript.

Plants

Seed stocks	*Report on the source of all seed stocks or other plant material used. If applicable, state the seed stock centre and catalogue number. If plant specimens were collected from the field, describe the collection location, date and sampling procedures.*
Novel plant genotypes	*Describe the methods by which all novel plant genotypes were produced. This includes those generated by transgenic approaches, gene editing, chemical/radiation-based mutagenesis and hybridization. For transgenic lines, describe the transformation method, the number of independent lines analyzed and the generation upon which experiments were performed. For gene-edited lines, describe the editor used, the endogenous sequence targeted for editing, the targeting guide RNA sequence (if applicable) and how the editor was applied.*
Authentication	*Describe any authentication procedures for each seed stock used or novel genotype generated. Describe any experiments used to assess the effect of a mutation and, where applicable, how potential secondary effects (e.g. second site T-DNA insertions, mosiacism, off-target gene editing) were examined.*
